# Exposed Pulp and Treatment

**Published:** 1883-05

**Authors:** C. R. Rowley

**Affiliations:** Niles, Mich.


### Exposed Pulp and Treatment.

BY DR. C. R. ROWLEY, NILES, MICH.

[Read before the Michigan State Dental Society.]

GENTLEMEN.—I would like to ask your attention to the reading of a ten-minute paper upon the above named subject. In so doing I think it is with a full realization of the fact, that this subject has been brought before this and other dental societies times without number. Notwithstanding the thought and discussion this subject has previously received, I believe you will bear me out in the assertion that, as a point in dentistry, it stands without parallel—the least understood of any found in everyday practice.

Tooth or dental pulp, as defined by Robley Dunglison, is a pultaceous substance, of a reddish gray color, very soft and sensitive, which fills the cavity of the teeth, and is well supplied with capillary vessels. We accept this short definition of dental pulp as the best given by any lexicographical work we have been permitted to examine, and yet we could wish for a better.

Old practitioners in the profession, this society favorably represents, have been taught two things directly relative to exposed pulp. The first one let me mention is: That this pultaceous substance, so called, has been known to live through a period of years, exposed to the attacks of hot and cold drinks, strong medicines, hard-crust intrusions, and tooth pick thrusts, and in rare cases the pulp has been known to resist the action of cobolt, and, like Richard, cry "for a horse that the battle may continue!" We older members of dentistry have often witnessed the above, and, on the other hand, we have seen the valiant knight of oral fame give up the ghost in a few days under the shadow of an oxychloride capping.

Now, gentlemen, we query : Can this pulp, known to be a tissue of uncommon vitality, be made to retain its life-flowing current under shield of foreign substance? My practice the past year encourages me to answer—Yes!

I will ask your attention while I mention four stages of pulp exposure, and trust you will fully understand my mode of treatment, and the four stages I will mention will be quite sufficient for all cases that come under our care.

The first pulp exposure possible (save that of fractured teeth) is caused by careless excavating or engine drilling. The shock to patient is severe, and not readily forgotten, and should lose the operator his patient. The trouble is of little moment, however, as the exposure can be bridged over with gold, and trouble rarely ensues.

Second stage requires skill and careful manipulation. Upon examination we find the pulp protected by many layers of partially decalcified dentine. If this substance under the excavator seems unresisting and apparently holding lime salts in solution, it is better to leave it in place to assist us in capping. We apply the rubber dam, dry the cavity, make application of eucalyptus oil, and glycerine, equal parts. Eucalyptus oil is used for its antiseptic properties, glycerine to guard against eschar. Cut back to strong enamel, excavate carefully the decomposed dentine about the free margin of the cavitv. In case pain ensues use remedies you are most familiar with for odontalgia. Now, cut disk of tin large enough to cover the entire surface of pulp chamber, press the disk carefully to place with a pellet of spunk moistened with olive or any flavored oil. Hermetically seal to place with oxychloride, using only enough of the zinc to accomplish that purpose. Now cover the oxychloride (much as the cavity will allow and leave space for filling material) with gutta-percha—the common pink base plate preferable—and in order to manipulate this rather unyielding substance it is better to cut into small blocks, and pack to place with smooth pointed pluggers.

We now pass to the stage of exposure where we find loose crumbs of thoroughly decalcified dentine floating about the cavity with each wave of saliva. Apply the dam, tie to place with silk floss, saturate a pellet of spunk with warm water, and cleanse the cavity. Absorb moisture with loosely rolled bibulous paper; now, with a ray of light thrown into the cavity with the mirror, we find the pulp largely exposed, differing in size of exposure as the size of tooth may differ. This degree of exposure calls into action creosote. Caution must be observed not to use creosote full nor one-half strength, as its powerful escharotic properties, working upon the epithelium of the pulp, will oftentimes congest and destroy what we are striving to protect and keep alive. Creosote diluted with glycerine, 1 and 4, I have used successfully. Leave the pellet containing creosote and glycerine in the cavity while you prepare the disk of tin, which can be done in two minutes or less. Where the rubber dam punch is used for cutting disk, tin No. 60 should be used. Place the disk on a piece of spunk and make it concavo-convex by pressing it with the oval end of an excavator. We must now have a solution of copal in chloroform, and fill the concavity of the disk with this solution. Place the filled side of the disk immediately over the pulp, and allow time for setting, usually about four or five minutes. Now flow over the disk oxychloride mixed to a consistency of maple syrup by using a little warm water in its mixture. Again, allow time for hardening of zinc, and then fill as in stage No. 2. My last stage of exposure I will mention is where we find life and death in the same tooth. There are times in the upper molars, also the lower, where we find the canal partially filled with dead pulp and nerve tissue, and a large degree of vitality in the remaining portion. We will speak only of the upper, where death ensues in the palatine root invariably, when the above is the case, caused undoubtedly in a measure by the action of foreign substances against the fang. Even in this case we would advise cleansing the palatine canal, and its filling with oxychloide, and the cap of tin, zinc, and gutta-percha, used as in stages 2 and 3.

Gentlemen, I have hurriedly placed these few thoughts together and I hope you will excuse incompleteness.

